# Scale-out of a *Total Worker Health®* approach for designing interventions to reduce teacher stress: pilot implementation evaluation

**DOI:** 10.1186/s12889-022-13241-6

**Published:** 2022-04-23

**Authors:** Lisa M. H. Sanetti, Alexandra M. Pierce, Lauren Gammie, Alicia G. Dugan, Jennifer M. Cavallari

**Affiliations:** 1 Neag School of Education, Department of Educational Psychology, University of Connecticut, Storrs, CT 06269 USA; 2 School of Medicine, Department of Medicine, Division of Occupational and Environmental Medicine, University of Connecticut Health Center, Farmington, USA

**Keywords:** Teacher well-being, Fidelity, Scale-out, *Total Worker Health*®

## Abstract

**Background:**

Teachers have high rates of daily stress and the majority of available interventions are focused at the teacher-level. Yet, best practices in *Total Worker Health*® approaches indicate organization-level interventions identified using a participatory approach are most effective. We conducted an exploratory scale-out pilot study to examine the adoption of the *Healthy Workplace Participatory Program* (HWPP), an evidence-based, *Total Worker Health* approach to engage employees (e.g., teachers) and supervisory personnel (e.g., administrators) in the design and implementation of workplace well-being interventions within two elementary schools.

**Methods:**

We evaluated the program both quantitatively and qualitatively collecting implementation outcome data (i.e., fidelity, acceptability, understanding, feasibility, system alignment) as well as data-driven adaptations using the Framework for Reporting Adaptations and Modifications-Expanded. Data from the first school informed scale-out adaptation of the HWPP intervention, HWPP-Education, within the second school. We compared implementation outcomes between Pilot Schools 1 and 2 to evaluate improvements in the adapted HWPP.

**Results:**

Adaptations to HWPP program content and process were suggested to increase feasibility and contextual fit. Acceptability, understanding, and feasibility ratings showed statistically significant improvements comparing School 1 to School 2 which implemented the improved HWPP-Education. Furthermore, users reported adaptations including shorter meeting design and faster process were feasible within their work context.

**Conclusion:**

This pilot study is the first attempt to scale out the HWPP to educators, and while not intended to confirm efficacy, it showed promising results for scale-out. Results from Pilot Schools 1 and 2 suggest systematic use of quantitative and qualitative implementation data can effectively inform scale-out efforts that increase critical outcomes such as fidelity, acceptability, understanding, feasibility, system alignment, and leader engagement as well as decrease the extent of system resources needed. As such, this scale-out process may be a feasible approach on which to base large-scale implementation efforts of the HWPP among educators.

## Background

Teacher stress has been defined as a teacher’s experience of unpleasant emotions that result from aspects of their work, particularly when their work demands exceed their available resources or ability to cope [1–4]. Surveys have found that teachers are tied with nurses in having the highest rates of daily stress among occupations, with nearly 59% of respondents reporting significant stress at least several days per week [5, 6]. Teachers’ chronic stress results in negative outcomes for both teachers and their students [7]. Chronically stressed teachers are less likely to use evidence-based classroom management and instructional strategies, more likely to experience physical and psychological health problems, and more likely to leave the field of education prior to retirement [8, 9]. Students of chronically stressed teachers are more likely to demonstrate disruptive behaviors [10], higher suspension rates [11], and increased salivary cortisol levels – an indicator of hypothalamic-pituitary-adrenal axis activity associated with the stress response [12].

Interventions targeting teacher stress have historically been grouped into four categories with a focus on individual (teacher) level changes. First, knowledge-based interventions (KBIs), which encompass various types of informational trainings, such as educating teachers about problem behaviors or providing psychosocial education on the risks associated with stress [13, 14]. Second, cognitive-behavioral interventions, which combine behavioral practice and cognitive training to provide teachers with knowledge and skills to manage job-related stress [15]. Third, mindfulness-based interventions, which utilize cognitive and behavioral strategies to shift the focus from thought content to the process of feeling and thinking by prioritizing awareness and non-judgmental acceptance, specifically targeting the symptoms of stress [16]. Fourth, behavioral interventions, which primarily focus on promoting specific skills and strategies to reduce teacher stress [17]. The majority of interventions focus on secondary prevention, aimed at modifying an individual’s response to stressors rather than primary prevention, reducing exposure to stressors.

Results of a systematic review of teacher stress-reduction interventions indicate that, across categories, interventions resulted in small-medium effect sizes [15]. These results suggest interventions focused on individual teachers can effectively address well-being issues at the individual level. Yet, available data indicate many sources of teacher stress are at the school or system (i.e., workplace) level [18]. As such, workplace-level health and wellness approaches may also be needed to address the teacher stress epidemic effectively, efficiently, and comprehensively [8].

Reviews of occupational health research support this conclusion; a combination of organizational and individual-focused approaches to job-stress intervention are more effective than either individually as they emphasize primary prevention (i.e., elimination or reduction in factors that give rise to teacher stress [19];). Results of a systematic review of organization-level interventions to address work-related stress in teachers, however, indicated the three reviewed interventions (i.e., changing task characteristics, coaching, and performance bonus + job promotion + mentoring) were minimally effective [20]. These results may not be surprising however, as all the organization-level interventions reviewed were top-down, one-size-fits-all approaches; as such, they were not fully aligned with evidence-based *Total Worker Health*® practices.

The National Institute for Occupational Safety and Health (NIOSH) *Total Worker Health* (TWH) approach recognizes work is a social determinant of health; workplace factors such as organizational policies/practices, work schedules, colleague relationships, and leadership affect worker health and well-being [21]. Available data on TWH approaches indicate (a) prevention-oriented, system-level interventions can be more efficient and effective than individual-level interventions alone; and (b) the most effective interventions are those that incorporate the unique input of end users [19, 22]. Systems-level interventions and participatory approaches are core components of a TWH approach [21, 23, 24]. Occupational health data indicate efforts to decrease teacher stress will be more effective and efficient if they include a school-level approach and include direct involvement of teachers in intervention planning and design.

The Center for the Promotion of Health in the New England Workplace (CPH-NEW), a NIOSH Center of Excellence for *Total Worker Health®*, developed the Healthy Workplace Participatory Program (HWPP), an evidence-based process that engages employees and supervisory personnel in the collaborative, iterative design and implementation of workplace-level health and wellness interventions [25].

The HWPP has effectively increased employee health and well-being across numerous worksites (e.g., property management companies, non-profit agencies, state government agencies, state prisons [25];, but until recently had not been implemented in schools. Given the critical need to reduce teacher stress and the promise of addressing root causes of teacher stress at the organizational level using a participatory approach, we conducted scale-out pilot evaluations of the HWPP in schools. Scaling-out is an approach to adapting and delivering evidence-based interventions to new populations or delivery systems [26]. More specifically, scaling-out is defined as “the deliberate use of strategies to implement, test, improve, and sustain [interventions] as they are delivered in novel circumstances distinct from, but closely related to previous implementations” ([26], p. 2).

In scaling-out, it may be possible to use the identical intervention (e.g., HWPP) with new populations or in a new delivery system; however, interventions often require adaptation to increase contextual fit with the population or delivery system [27]. Adaptation, in the context of interventions, is defined, as “a process of thoughtful and deliberate alteration to the design or delivery of an intervention with the goal of improving its fit or effectiveness in a given context” ([28], p. 1). When adaptation is required, it is essential to identify and retain the core elements of the intervention and modify, eliminate, or add peripheral intervention elements that complement, but do not conflict with, the core components [26].

Implementation outcomes, defined as, “the effects of deliberate and purposive actions to implement new [interventions]” ([29], p.65) include fidelity, the extent to which an intervention is delivered as intended (including dosage, exposure, adherence), but also acceptability, appropriateness (e.g., system alignment, understanding), feasibility, adoption, costs, penetration and sustainability [29]. Increasingly in education research, fidelity data are reported to document adherence to intervention steps, supporting the internal validity of intervention evaluation studies [30]. However, implementation outcome data also can be used to inform the adaptation of evidence-based interventions being scaled-out to education from another field or within education to a new age-range (e.g., elementary to secondary) or delivery method (e.g., in-person to online [31];). That is, implementation outcome data from a pilot trial within a new field, setting, or delivery method can inform data-driven intervention adaptation for a subsequent pilot trial [31]. This approach allows for data-driven, systematic intervention adaptation that can facilitate use of already developed, effective interventions across fields, settings, and delivery methods, greatly expediting the delivery of evidence-based services across human service sectors.

To facilitate systematic evaluation of adapted evidence-based interventions, it is essential to document data-driven intervention modifications and the adaptation process [28]. Stirman and colleagues [28] have developed and refined the Framework for Reporting Adaptations and Modifications-Enhanced (FRAME), which includes documentation of (a) when in the implementation process the modification was made, (b) whether the modification was planned/proactive or unplanned/reactive, (c) who participated, (d) the goal of the modification, (e) the reasons for the modification, (f) what was modified, (g) at what delivery level the modification was made, (h) the nature of modifications, and (i) the extent to which the modification was fidelity-consistent. This proactive and systematic documentation of adaptations facilitates replication and comparison across intervention evaluations in research or practice.

We sought to better understand how to scale-out the HWPP Design Team process to elementary schools. This research had three aims. The first aim, addressed with elementary school, Pilot School 1, was to utilize implementation outcome data (i.e., fidelity, acceptability, appropriateness, feasibility; see Table 1) to identify adaptations to the HWPP Design Team process that may facilitate scale-out of the HWPP to elementary school, Pilot School 2. The second aim, addressed between pilot schools, was to document implementation outcome data-driven adaptations to HWPP using the FRAME to inform implementation within Pilot School 2. The third aim, addressed in Pilot School 2, was to evaluate whether the adapted HWPP-Education improved Design Team process implementation outcomes while maintaining core components of HWPP.

**Table 1:** The Healthy Workplace Participatory Program IDEAS Process

IDEAS Step and Objective	Goal	Activities
***Intervention Design Phase – Design Team Primary Role***		
*Step 1: Identify problems and contributing factors*	Identify the root causes of a top health and wellness concern by generating a list of factors that contribute to or cause the concern	• The “All Employee Survey” that measures physical and psychological health (e.g., sleep, stress), interpersonal relationships (e.g., leadership, affiliation, role conflict), and work-related factors (e.g., autonomy, workload) is used to gather data on employee concerns, all employees complete. • Employee input can also be obtained through focus groups using HWPP-specific focus group protocols. • Design Team members (a) review the All Employee Survey results, focus group result, or both; (b) write their top three concerns individually on post-it notes; and (c) place the post-it notes on a flip chart. • The facilitator guides the Design Team through grouping concerns into related concerns. • Once all areas of concern are identified, then each Design Team member is given three dot stickers, which they use to vote for their top concerns. Design Team members place each dot sticker on the flip chart near the area(s) of concern they want to vote for as most important. Each member may distribute their dot stickers however they would like (e.g., all three stickers on one concern, one sticker for three different concerns). • The Design Team (a) reviews voting results, (b) identifies the top priority concern, and (c) creates a fishbone diagram to map the root causes of the concern.
*Step 2: Develop intervention objectives and activities*	Using the information from the root cause analysis, identify solutions that result in full or partial resolution of the top concern.	• The Design Team develops an objective that represents a realistic and meaningful improvement (e.g., reduce the number of teachers indicating low levels of personal accomplishment). • The Design Team brainstorms solutions (e.g., increased structured public and private noticing of accomplishments) and specific activities (e.g., daily recording of personal “wins,” staff “shout outs” submitted to office and announced to whole school daily) to enact each solution. The facilitator records all solutions and activities.
*Step 3: Set selection criteria*	Identify criteria, or key performance indicators, to consider when evaluating any intervention.	• Led by the facilitator, the Design Team determines what (a) the scope of the intervention should be (e.g., how many employees will it reach?), (b) measurable benefits should result from the intervention, (c) resources are available for the intervention, and (d) possible barriers to the intervention exist. Interventions that meet most or all selection criteria are deemed to have the greatest likelihood of success.
*Step 4: Form interventions*	Group multiple solutions to form interventions, and then rate and prioritize three intervention options that can be considered for adoption by the Steering Committee.	• The Design Team reviews brainstormed solutions and associated activities and groups them into multi-component interventions. These interventions may be distinct from one another, or the Design Team may choose to present a “basic essentials” intervention, a fully comprehensive intervention, and an intervention that includes the basic essentials and a few additional activities.
*Step 5A: Rate interventions*	Rate the three prioritized intervention options using each key performance indicator.	• The facilitator leads the Design Team in analyzing each intervention against the key performance indicators (i.e., scope, benefits, resources, barriers) identified in Step 3. • The Design Team rates each intervention as low, medium, or high on each indicator.
***Intervention Implementation Phase – Steering Committee Primary Role***		
*Step 5B: Rate and select interventions*	Design Team presents their work to the Steering Committee who rates and approves the intervention(s) to be implemented.	• The Design Team presents the interventions to the Steering Committee and answers questions about their process and resulting interventions. • The Steering Committee rates each intervention as low, medium, or high on scope, benefit, resources, and obstacles. The Steering Committee may provide feedback to the Design Team and request further revisions to the interventions or select the intervention(s) as they are presented.
*Step 6: Plan and implement interventions*	The Steering Committee develops an implementation plan for the selected intervention(s).	• It is generally recommended that the Steering Committee prioritize the sequence of intervention activities, identify personnel who should be involved, provide training if needed, and develop a communication plan. Few resources and little guidance are provided in the IDEAS Tool for specific actions at this step, as the intervention planning and implementation will be unique to each context and guided by implementation science.
*Step 7: Monitor and evaluate intervention activities*	Collect data on intervention implementation and effectiveness.	• The IDEAS tool provides few resources and little guidance for specific actions in this step, as evaluation will be unique to each intervention and implementation context.

## Methods

### Intervention: the healthy workplace participatory program

The HWPP is an evidence-based approach to engage employees (e.g., teachers) and supervisory personnel (e.g., administrators) in the design and implementation of workplace well-being interventions. The HWPP process includes a Design Team made up of front-line workers (e.g., teachers) and a Steering Committee composed of management or other leaders (e.g., principals, pupil service personnel). The core of the HWPP process is the Intervention Design and Analysis Scorecard (IDEAS) process, which is divided into two phases (see Table 1). In the first phase, the Design Team engages in a five-step structured process to (a) identify problems and contributing factors, (b) develop intervention objectives and activities, (c) set intervention selection criteria, (d) form interventions, and (e) rate interventions using selection criteria. In the second phase, the Steering Committee engages in a three-step process to (a) rate and select interventions, (b) plan and implement interventions, and (c) monitor and evaluate interventions (see Table 1). The focus of this scale-out pilot is limited to the five steps of the Design Team process because these steps represent the unique, structured, participatory process by which workplace-level, and workplace-specific TWH interventions are designed (see Table 1). The latter steps by the Steering Committee are akin to adoption, implementation, and evaluation of *any* school-based intervention effort; they are not unique to the HWPP.

### Participants

Two elementary schools served the basis for the pilot evaluation. Pilot School 1 is located in large suburban district in the Northeastern United States and serves students in grades 3–5. Pilot School 1 consisted of 396 students, 29 general education and specials teachers, three special education teachers, 11 paraprofessionals, two pupil services personnel, and two school-level administrators. Of the students, 88.4% identified as White, 7.0% as Black or African American, 2.3% as Hispanic/Latino, and 2.3% as two or more races. At the time of the study, 46.4% of students were eligible for free- or reduced-price lunch.

Pilot School 2 is located in large suburban district in the Northeastern United States and serves students in grades K-5. Pilot School 2 consisted of 273 students, 24 general education and specials teachers, 2 special education teachers, 11 paraprofessionals, 4 pupil services personnel, and one school-level administrator. Of the students, 39% identified as White, 31% as Hispanic/Latino, 15% as Black or African American, 9% as two or more races, and 6% as Asian. At the time of the study, 84.1% of students were eligible for free- or reduced-price lunch.

For Pilot School 1, the first author met with the school administration in the summer prior to the 2018–2019 academic year to discuss the pilot study. The district- and building-level administrators were supportive, and the principal offered time during the September faculty meeting for the first author to introduce the study to the teachers. Teachers interested in being on the Design Team indicated their interest and the first author followed up with them individually to answer questions and obtain consent. Recruitment in Pilot School 2 followed the same procedures with the first author meeting with the school administration in Fall 2019.

### Implementation of the HWPP elements

#### HWPP all employee survey

The All Employee Survey (AES) was designed by CPH-NEW to provide organizations with an overall assessment of employee attitudes related to health, safety, and wellness and is meant to support the IDEAS process [32]. The 122-item survey takes approximately 30 min to complete. The survey provided feedback on (a) issues related to physical work environment; (b) interpersonal and social interactions that support or detract from a healthy worksite culture; and (c) employee perceptions of their health and well-being.

The AES was administered by the research team via Qualtrics and emailed to all teachers. Responses were anonymous. In Pilot School 1, teachers brought a laptop or tablet to a faculty meeting and had 35 min to complete the survey. The response rate was 98%; one teacher who was absent did not complete the survey. In Pilot School 2, teachers had 2 weeks to complete the survey on their own time; two reminder emails from the research team were forwarded to teachers by the principal. The response rate was 80%, with representation from all grades.

#### IDEAS process

The HWPP Design Team Facilitator Manual was utilized to facilitate the Design Team IDEAS process in Pilot School 1 [33]. The manual included (a) an overview of the TWH approach and the HWPP, (b) outlines for facilitating eight Design Team meetings (see Table 2), and (c) Design Team Member Notebook pages that included IDEAS worksheets for use during meetings. The meeting activities followed the HWPP as outlined in Tables 1 and 2. In Pilot School 2, the adapted HWPP-Educator (HWPP-E) included a manual to facilitate the Design Team IDEAS process as outlined in Table 2.

**Table 2:** Alignment of HWPP Components across Sessions for Schools 1 and 2

HWPP Components^a^ in School 1		IDEAS Step	HWPP-E Components for School 2	
Sessions 1–8	Introductions, Introductory questions		Sessions 1–5	Introductions kept for Session 1; removed for all other sessions.
Session 1	Reflection on TWH	Start-up 1	Session 1	Removed
	Definition of TWH			Brief discussion
	HWPP roles			Brief discussion
	HWPP processes and IDEAS tool			Brief discussion
	Ground Rules			Maintained
Session 2	Brainstorming on health, safety, wellbeing in workplace	Start-up 2		Removed
	Ideal workplace brainstorming			Removed
Session 3	Identify top 3–4 health and safety concerns individually	Start-up 3		Kept
	Group concerns by theme	Start-up 3		Kept
	Vote to identify top priorities	Start-up 3		Kept
Session 4	Root Causes Analysis	Step 1	Session 2	Kept
Session 5	Set Measurable Objective	Step 2	Session 3	Kept
	Brainstorm Solutions	Step 2		
Session 6	Establish Criteria for Evaluating Interventions	Step 3	Session 4	Revised to a brief discussion
Session 7	Create 3 intervention options	Step 4		Kept, but number of interventions not specified
	Apply selection criteria to solution activities	Step 5A		Revised to describe the scope, benefits, resources
Session 8	Present to Steering Committee	Step 5B	Session 5	Revised-presented to Principal; identified SC members based on intervention options selected

^a^Per August 2017 version of HWPP Facilitator Manual

### Scale-out data collection

Five implementation outcomes (fidelity, acceptability, understanding, feasibility, and system alignment) were measured in both pilot schools. Outcome and measure alignment are illustrated in Table 3.

**Table 3:** Alignment of Implementation Outcomes and Measures

Implementation Outcome	Measure(s)
Fidelity	Dosage: Duration and frequency of meetings Exposure: Extent of meeting attendance of Design Team members Adherence: % of IDEAS Process Steps 1–5 completed
Acceptability	URP-IR Acceptability subscale
Understanding	URP-IR Understanding subscale
Feasibility	URP-IR Feasibility subscale
System Alignment	CPH-NEW Process Evaluation URP-IR System Climate subscale URP-IR System Support subscale

#### HWPP Fidelity

The first author facilitated and the second author attended all HWPP meetings. To gather dosage and exposure data, at the beginning of each meeting, the facilitator recorded the date of the meeting, the start time, and all Design Team members in attendance; at the conclusion of each meeting, the facilitator recorded the end time and noted if any members left the meeting early. To record adherence, throughout the meeting, the facilitator adhered to the HWPP Design Team Facilitator Manual, checking off meeting components on the CPH-NEW-provided meeting outlines. If the facilitator missed any components, the second author prompted the facilitator to ensure all components were delivered in the prescribed order. In Pilot School 2, the revised HWPP-E Guide was used and a member of the research team prompted the facilitator to ensure all components were delivered in the prescribed order.

#### Usage rating profile-intervention (URP-IR)

To assess the social validity of the HWPP process and the resulting interventions, Design Team members completed the URP-IR [34] to provide feedback on the HWPP process. The URP-IR is a 29-item, 6-point Likert scale (1 = *Strongly Disagree* to 6 = *Strongly Agree*) questionnaire that includes items related to (a) acceptability, (b) understanding, (c) home-school collaboration, (d) feasibility, (e) system climate, and (f) system support. The three items related to the home-school collaboration subscale were omitted, as they were not relevant to the current study; thus, participants completed 26 items. The subscales demonstrate acceptable internal consistency reliability (Cronbach’s alpha range from .72–.95 [35];).

#### Process evaluation rating sheet

At the end of the study, Design Team members completed the HWPP Process Evaluation Rating Sheet [36]. This is a 12-item, 5-point Likert scale (1 = *Strongly Disagree* to 5 = *Strongly Agree*) questionnaire that includes three subscales (4 items each) relevant to the Design Team process and adaptation (i.e., Organizational Support and Engagement, Design Team Engagement, Program Facilitation Effectiveness). Items are summed within subscales for possible score ranges from 4 to 20.

#### Design team focus group guide

The research team developed a focus group guide to obtain qualitative data specific to the Design Team process with the following four prompts: (a) What are your overall impressions of the Design Team process and materials? (b) What would you keep the same? (c) What would you change (add, omit, modify) to make the Design Team process more efficient, feasible, and effective? (d) Is there anything else you’d like to share? In Pilot School 1, all Design Team members engaged in a 1-h audio-recorded focus group in a classroom after school with the first author at the end of the study. All members engaged in dialogue and responded to all four prompts. In Pilot School 2, we were able to complete all Design Team processes, but due to COVID-19 school closures, we were unable to complete a typical in-person focus group. Instead of an in-person focus group, Design Team members were contacted individually via email to provide responses to the same questions used in the Pilot Study 1 focus group.

### Data analysis

Descriptive data are presented for implementation outcomes. ANOVA was used to compare implementation data by school. Given the exploratory nature of the pilot study, the six-phase approach to inductive thematic analysis was applied to analyze focus group data [37]. The first author (a) read the focus group transcript carefully three times to ensure familiarity; (b) coded data using descriptive labels to identify relevant data features; (c) grouped similar codes together to identify themes; (d) compared potential themes to the entire data set to verify representativeness and revise as needed; (e) named and defined the final set of themes; and (f) synthesized results to summarize findings. To increase credibility, another research team member who was not involved in the focus group or initial analysis reviewed the transcripts and the initial findings [38].

## Results

We followed an iterative process of adaptation where data from Pilot School 1 informed the adaptation of the HWPP before implementation in Pilot School 2. Therefore, data from Pilot School 1 is presented followed by data from Pilot School 2.

### Pilot school 1

#### Design team participants

Seven teachers in a primary school (grades 3–5) located in a large suburban town in Connecticut volunteered to participate on the Design Team. The teachers represented all grades in the school. Six teachers were Caucasian, one was Black. Five were general education classroom teachers, one was a special education teacher, and one was the music teacher. Six teachers identified as female, one identified as male. On average, they had 11 years teaching experience (range: 4–20).

#### HWPP design team process

The seven Design Team members agreed to meet weekly for 2 h in a classroom after school to complete the HWPP Design Team process. Using the HWPP facilitator guide, the facilitator provided all materials necessary to complete the three “Start-Up” sessions and IDEAS Tool Steps 1–5. Specifically, the research team aggregated and graphed de-identified AES survey data. The Design Team used these data to inform their selection of a top concern, which was stress/burnout. Upon identifying the top concern per HWPP processes, the Design Team determined the five major contributing factors to stress were (a) poor communication, (b) lack of self-care, (c) high workload, (d) inadequate resources, and (e) managing student behavior. Based on this, the Design Team proposed a total of eight possible interventions for consideration by the Steering Committee. Three interventions targeted staff physical and psychological well-being: (a) opportunities for physical activity before or after the school day, (b) a presentation by a Human Resources staff member to review health and wellness programs and resources available to staff, and (c) staff training on stress management strategies. Five of the proposed interventions targeted improvement of the workplace climate: (a) increasing staff members’ voice at meetings, (b) restructuring the schedule to allow teachers to have brief breaks, (c) providing positive feedback notes to staff, (d) increasing professionalism by limiting conversations about students to individuals with a “need to know,” and (e) ensuring teachers received minutes from advisory meetings (i.e., monthly meeting for teachers to bring up concerns to administration).

#### Implementation results

##### Fidelity

**Fig. 1:**
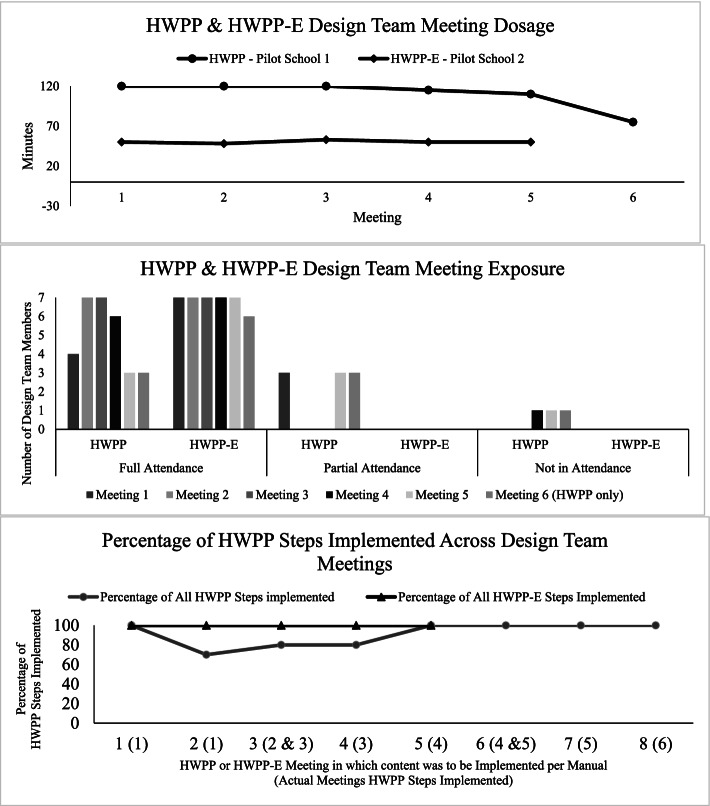
HWPP and HWPP-E Fidelity Data

Three dimensions of fidelity were gathered throughout the Design Team process to inform adaptation: dosage, exposure, and adherence (Table 2). With respect to dosage, per the HWPP Design Team Facilitator Manual, each of the eight meetings should last approximately 2 hours. As such, Design Team members consented to up to eight, 2-h weekly meetings. In facilitation of the Design Team meetings, however, it became evident that it would not take 16 h for the Design Team to complete Steps 1-5A of the HWPP. The Design Team met six times after school for an average of 1 h and 50 min (range: 75–120 min) with duration declining across meetings (see Fig. 1 top graph). Although the group attempted to meet weekly, the six meetings occurred over ten school weeks due to school breaks, meeting conflicts, and inclement weather closings. In terms of exposure, Design Team members were expected to attend the full duration of all meetings; however, this was not the case for most meetings (see Fig. 1 middle graph). Reasons for missing meeting time included challenges finding the meeting location, competing professional or personal responsibilities.

Overall, the facilitator’s self-ratings indicate high levels of adherence to specific steps outlined in the Design Team Facilitator Manual (see Fig. 1 bottom graph). Specially, all core intervention steps were implemented across meetings. In the second meeting, it became evident that a full 2 h would not be needed to complete the content for Start-Up Meeting 2. In consultation with the Design Team members, it was decided to continue for 2 h and move forward with the content; IDEAS Steps 1-5A, designed to be implemented in separate meetings, were combined at times (see x-axis of the bottom graph in Fig. 1). As a result, some intervention components that were supposed to occur in Sessions 2–4 (e.g., reviewing ground rules, previewing next meeting) were not implemented as planned because they were not necessary as their purpose was as a reminder of the last meeting or preview of the next meeting.

##### CPH-NEW process evaluation ratings

At the end of the study, Design Team members completed the CPH-NEW Process Evaluation Rating Sheet [36] to assess system alignment (Table 4). The highest mean (SD) ratings were observed for Program Facilitation Effectiveness 18.8 (1.79), followed by Design Team Engagement 17.0 (3.32) and Organizational Support and Engagement was 13.4 (6.15) (Table 4).

**Table 4:** CPH-NEW Process Evaluation Ratings by School

Subscale^a^	School 1 Mean (SD)	School 2 Mean (SD)	ANOVA *p*-value
Organizational Support and Engagement	13.4 (6.15)	17.1 (1.68)	0.18
Design Team Engagement	17.0 (3.32)	18.7 (1.11)	0.25
Program Facilitation Effectiveness	18.8 (1.79)	19.1 (0.90)	0.72

^a^ All items of the CPH-NEW Process Evaluation Rating were assessed on a 5-point Likert scale (1 = *Strongly Disagree* to 5 = *Strongly Agree*); each subscale includes 4 items for possible score range of 4 to 20

##### Usage rating profile-intervention (URP-IR)

At the end of the study, Design Team members completed the URP-IR [34] to assess Acceptability, Understanding, Feasibility and additional aspects of System Alignment (Table 5). Acceptability, Understanding, and System Climate all had mean (SD) ratings above 4 at 4.19 (1.13), 4.08 (0.79), and 4.15 (1.14), respectively (Table 5); Feasibility was lower at 3.88 (0.99). System Support, a reverse scored subscale, was lower at 3.67 (1.15), indicating the Design Team did not require significant system support.

**Table 5:** Usage Rating Profile-Intervention (URP-IR) by School

URP-IR Subscale^a^	School 1 Mean (SD)	School 2 Mean (SD)	ANOVA *p*-value
Acceptability	4.19 (1.13)	5.12 (0.72)	0.02
Understanding	4.08 (0.79)	5.67 (0.48)	0.002
Feasibility	3.88 (0.99)	5.33 (0.53)	0.003
System Climate	4.15 (1.14)	4.54 (0.56)	0.30
System Support	3.67 (1.15)	2.57 (0.68)	0.008

^a^ All items of the URP-IR were assessed on a 6-point Likert scale (1 = *Strongly Disagree* to 6 = *Strongly Agree*)

##### Design team focus group

At the end of the study, the Design Team members completed a semi-structured focus group to identify potential adaptations to the Design Team process. Six primary learnings resulted: (a) members were highly supportive of a participatory approach to addressing teacher health and wellbeing, (b) the core aspects of the Design Team process systematically built upon one another, (c) the content in the Start-up Sessions did not directly inform intervention development and noted that some activities, such as the Ideal Workplace, were demoralizing as they did not believe their ideal could be achieved; (d) the number and duration of meetings would be a barrier to many teachers and staff participating; (e) the materials for Design Team included too much information overall and on each page, which hindered understanding and engagement; (f) the latency from the beginning of the process to intervention implementation was too long.

### HWPP adaptations-FRAME

Utilizing the implementation outcome data described above, the research team adapted four core aspects of the HWPP Design Team process (IDEAS Tool Steps 1-5A). Table 6 documents all FRAME-recommended aspects of adaptation, as well as the Pilot School 1 data that informed each adaptation. All adaptations were systematically planned and made prior to implementation within Pilot School 2. Together, implementation outcome data consistently indicated time was an issue to address. Dosage data demonstrate a decreasing trend in meeting duration across time, while exposure data show decreasing levels of meeting attendance. Although the reasons for teachers being unable to attend Design Team meetings were reasonable, absences limited representation of each grade in the meeting and the ability to share information with and get feedback from grade-level colleagues between meetings. Perfect attendance is neither required nor expected of Design Team members, but adaptations to increase attendance are critical to optimizing the process. Focus group results indicated concerns with time required of participants and time to intervention, in alignment with fidelity data. Adherence data to core components was high across meetings but demonstrate real-time decisions to omit superfluous steps to streamline content and maximize use of meeting time. Further, Design Team focus group results highlighted the need for streamlined materials to facilitate keeping members on-task during meetings. As time is regularly noted as a barrier to intervention implementation, it is not surprising that all these adaptations were related to decreasing the time required. Specifically, the research team reviewed the IDEAS Tool Steps 1-5A and omitted aspects that did not contribute to identification of the top concern or identification of related interventions (see Table 6). As the Design Team indicated a desire to decrease latency to health and wellbeing interventions being adopted and implemented (IDEAS Steps 5B-7), we maintained a weekly meeting schedule. Further, because schools are flat organizations in which teachers regularly interact with one another (as compared to large organizations with multiple managerial levels and locations), we omitted many of the introductory or “ice breaker” activities at the beginning of meetings and many of the aspects of the “Start-Up” meetings. Further, teachers already have many skills that allowed us to modify portions of the process; for example, unlike other professions, teachers did not need instruction in how to write an objective and were able to do so in less than a quarter of the initially allotted time. Together, these changes decreased the amount of time needed to complete the IDEAS Tool Steps 1-5A from 8 2-h meetings to an estimated 5 1-h meetings. Based on these adaptations, the research team revised the HWPP Design Team Facilitator Manual to the HWPP-E Design Team Facilitator Guide.

**Table 6:** Summary of HWPP Adaptations and Modifications for School 2 based on Implementation Outcome Data from School 1

Who Participated	Goal	Rating Scales (Process Evaluation & URP-IR)	Design Team Focus Group Data	Study 1 Related Fidelity Data	What is modified	At what level of delivery	Nature of modification	Relationship to fidelity / core elements
Researchers	Improve fit with recipients	Total time required to implement the process was lowest rated item on URP-IR Feasibility subscale.	∙ Too many meetings. ∙ It took too long to identify and implement intervention. ∙ It was demoralizing to reflect on TWH and Ideal Workplace when not able to be obtained. ∙ It took too long to identify and implement a stress-reduction intervention.	Exposure: Design Team increasingly members skipped meetings or left early. Adherence: Data were variable for some meetings in which content did not take the projected amount of time.	Decreased number of meetings-combined 8 meetings into 5 (see Table 1). Maintained only core components of IDEAs process.	Group	∙ Removing elements ∙ Substituting content ∙ Shortening / condensing some components	Fidelity consistent
Researchers, Administrator	Increase retention & satisfaction	Total time required to implement the process was lowest rated item on URP-IR Feasibility subscale.	∙ The duration of meetings was not sustainable.	Dosage / Exposure: Design Team members increasingly skipped meetings or left early.	Decreased duration of meetings from 2 h to 1 h.	Group	∙ Shortening / condensing pacing	Fidelity consistent

### Pilot school 2

#### Design team participants

Six teachers and the school psychologist in an elementary school (grades PK-5) in a large suburban town in Connecticut volunteered to participate on the Design Team. The teachers represented all grades taught in the school. Five teachers and the school psychologist were Caucasian, one teacher was Black. Three teachers were general education classroom teachers, two were special education teachers, and one was the music teacher. All identified as female. On average, they had 14 years teaching experience (range: 8–24).

#### Design team process

The seven Design Team members agreed to meet weekly for 1 h in a classroom before school to complete the HWPP-E Design Team Process. The facilitator provided all HWPP-E materials necessary as in Pilot School 1. The Design Team identified improving the culture among staff as their top concern, with three major contributing factors: lack of (a) understanding of staff members’ roles, (b) feelings of being unsupported and disrespected, and (c) lack of use of teachers’ strengths. Based on this determination, the Design Team proposed a total of four possible interventions for consideration by the Steering Committee: (a) have all staff members (teachers by team) present their roles and responsibilities at a staff meeting; (b) be another staff member for an hour to increase understanding; (c) have one staff meeting a month run by staff and without the administrator, to improve staff relationships; and (d) read Teach with Your Strengths and complete StrengthsFinder assessment to help teams and administrator delegate tasks to people with aligned strengths.

#### Implementation results

##### Fidelity

As in Pilot School 1, three dimensions of fidelity were gathered throughout the Design Team process. With respect to dosage, per the HWPP-E Design Team Guide, each of the five meetings should last approximately 1 h. The Design Team met five times before school for an average of 50 min (range: 48–53 min) with duration consistent across meetings (see Fig. 1 top graph). With respect to exposure, Design Team members were expected to attend the full duration of all meetings. One member was not available for the fifth meeting, otherwise all members attended the full duration of all meetings (see Fig. 1 middle graph). Overall, the facilitator’s self-ratings indicate high levels of adherence to specific steps outlined in the HWPP-E Design Team Facilitator Guide (see Fig. 1 bottom graph). Specially, all core intervention steps were implemented as planned, suggesting the HWPP-E adaptations were appropriately distributed across five, 1-h meetings.

##### CPH-NEW process evaluation ratings

As before, system alignment was assessed using the CPH-NEW Process Evaluation Rating Sheet [36]. As before, the highest mean (*SD*) rating was observed for Program Facilitation Effectiveness at 19.1 (0.90), followed be Design Team Engagement at 17.0 (3.32) and Organizational Support at 17.1 (1.68) (Table 4). While all ratings were higher at Pilot School 2 as compared to Pilot School 1, the differences were not statistically significant.

##### URP-IR

As before, the Design Team members completed the URP-IR [34]. The mean (*SD*) ratings were above 5 for Acceptability, Understanding, and Feasibility at 5.12 (0.72), 5.67 (0.48), and 5.33 (0.53), respectively (Table 5). System Climate was above 4 at 4.54 (0.56) and System Support was below 3 at 2.57 (0.68). Statistically significant increases were observed between Pilot School 1 and Pilot School 2 ratings of the Acceptability, Understanding, and Feasibility, and a statistically significant decrease was observed for System Support (see Table 5).

##### Design team focus group

At the end of the Design Team process, members were asked to respond to the same questions as in Study 1. Seven primary learnings resulted: (a) the 1-h meeting duration was feasible and aligned with typical school committee meetings; (b) holding meetings before school helped participants focus, as there was a definite end time; (c) although members would have liked to move through process faster, they agreed meeting weekly was feasible, whereas longer or more frequent meetings would not have been feasible; (d) the HWPP-E Design Team process allowed brainstorming, but also progress through steps; (e) all aspects of meetings were aligned with the core goal of identifying top concern and related interventions; (f) the latency from start of Design Team to presentation to Steering Committee was acceptable; and (g) the HWPP-E materials provided enough of an overview of each step and facilitated the in-meeting work.

## Discussion

We successfully implemented and adapted a systems-level intervention to address teacher stress in two pilot schools. The HWPP fills an important need for educators by providing an evidence-based, participatory approach to addressing workplace-level health and wellness concerns (Robertson et al., 2013). The purpose of this exploratory pilot study was to engage in a data-driven scale-out of HWPP to schools.

Results of Pilot School 1 demonstrated concerns related to exposure, dosage, and adherence based on the original HWPP intervention. Attendance at Design Team meetings declined over time, with members missing or partially attending meetings. This resulted in insufficient dosage and exposure to core components of the intervention. Further, social validity data demonstrated that the feasibility of the HWPP intervention was a barrier to implementation. The lowest rated item on the URP-IR [34] Feasibility subscale was, “The total time required to implement the intervention procedures would be manageable.” In focus groups, Design Team members reported concerns about the number of meetings required and the latency between Design Team meetings and intervention implementation. The Design Team also reported that some activities were demoralizing (i.e., because desired changes were unattainable) or unnecessary. Last, focus groups indicated that the duration of Design Team meetings was a threat to sustainability. These results indicated a clear need for systematic adaptations to the HWPP intervention for the second scale-out pilot study. These results are consistent with the ample documentation of mismatch between interventions and novel application in new communities or service delivery systems [27] and calls to systematically identify and characterize adaptations [27, 28].

FRAME was used to document the data-driven, fidelity-consistent adaptations made to the HWPP to improve contextual fit with intervention recipients, increase retention, and improve satisfaction. The number and duration of meetings was modified by maintaining only the core components of the IDEAS process, eliminating many aspects of the three recommended start-up meetings (i.e., surface components), and shortening other aspects in which teachers had strong skills (e.g., objective writing), resulting in five 1-h meetings. The modified intervention (HWPP-E) was implemented in Pilot School 2 to determine if adaptations improved implementation outcomes. The FRAME is not regularly used to document adaptations in education, but the adaptation of the HWPP demonstrates its utility in scaling out an intervention.

With regard to dosage, URP-IR [34] data demonstrate the modifications made to the duration and number of meetings significantly improved the feasibility and acceptability of the intervention. With regard to exposure, attendance across HWPP-E meetings was consistent at 100% of participants for all but one meeting for which one member was absent. Adaptations made to the intervention allowed for adequate dosage and exposure to key intervention components in Study 2. With regard to adherence to HWPP-E intervention, components also increased to 100% across all meetings. Beyond fidelity, URP-IR results indicate significant improvements in Understanding of HWPP and decreases in the need for Systems Support to implement HWPP. Overall, the data-informed, fidelity-consistent adaptations, documented using FRAME, effectively improved intervention implementation and increased social validity. These changes may increase sustainability of HWPP, designed to be an on-going approach to improving TWH outcomes.

Ratings from both the URP-IR and CPH-NEW Process Evaluations indicated improved organizational engagement and reduced need for system support after adaptation of the HWPP. Specifically, the CPH-NEW process evaluation results indicate an increase in school leaders’ engagement and commitment with the adapted HWPP, which is consistent with the defining elements of a TWH approach [21]. As such, the scale-out adaptation of the HWPP may have increased the program’s alignment with a TWH approach within this work sector.

### Implications for research and practice

There are two primary areas of implication for teacher mental health research and practice. First, results of these initial studies suggest the HWPP can be an effective participatory approach to identifying workplace-specific root causes of teacher stress and developing context-specific interventions. As such, the HWPP may serve as a primary approach to addressing teacher stress that could be combined with teacher-level interventions to deploy stress-reduction interventions more comprehensively, efficiently, and effectively at the systems-level. Second, this sequence of pilot studies showcases application of best practices in scaling-out an evidence-based intervention by (a) implementing the original intervention in a new setting, (b) using data to inform adaptations, (c) systematically documenting the adaptations using FRAME, and (d) evaluating the adapted intervention. There are evidence-based interventions developed, evaluated, and effective with certain age ranges within schools (e.g., elementary) that may be effective for a broader range of students (e.g., elementary and middle school). Systematically scaling-out these evidence-based practices, instead of developing new interventions, could accelerate the time to positive impact on educator and student outcomes.

The field of occupational health has identified the critical need for such studies to improve the dissemination and implementation of worker health interventions across diverse occupational settings [39]. Furthermore, there is benefit in using the TWH approach provided by the HWPP to improve worker well-being. Specifically for educators, where stress is the main threat to worker well-being, a TWH approach builds upon the traditional occupational health approach to also include work organization as well as consideration of work and non-work risk factors [40].

## Strengths and limitations

The current study strengths include the use of the HWPP, an evidence-based TWH approach, along with established implementation measures and a framework for guiding scale-out. Although the results from these initial exploratory scale-out pilot studies are promising, there are limitations that are important to consider. First, these pilot studies included a limited sample and setting. This is appropriate for pilot tests, yet it is likely the HWPP requires more or different adaptations for schools with different characteristics (e.g., size, age range, setting). Second, the first author served as the facilitator; it is possible the levels of fidelity would be different if implemented by an educator. CPH-NEW generally recommends, however, that an external facilitator model the initial IDEAS cycle, and then transfer to a workplace-based facilitator in subsequent cycles [33]. Third, psychometrically sound measures of fidelity have yet to be developed for HWPP, however the methods used were similar to those commonly used in school-based research and practice [30]. This pilot study was not intended to confirm the efficacy of the interventions designed and implemented by the educators to reduce stress and improve well-being. Additional studies would be required to assess whether the interventions developed and implemented as part of the HWPP-E were effective in reducing stress among teachers.

## Conclusion

Results from Pilot Schools 1 and 2 suggest systematic use of quantitative and qualitative implementation data can effectively inform scale-out efforts that increase critical outcomes such as fidelity, acceptability, understanding, feasibility, system alignment, and leader engagement as well as decrease the extent of system resources needed. As such, this scale-out process may be a feasible approach on which to base large-scale implementation efforts of the HWPP among educators.

## Data Availability

Due to the privacy of the participants, the dataset generated and analyzed during the study is not publicly available. The data that support the findings of this study are available from the corresponding author, [LS], upon reasonable request.

## Abbreviations

AES: All-Employee Survey
ANOVA: Analysis of Variance
CPH-NEW: Center for the Promotion of Health in the New England Workplace
FRAME: Framework for Reporting Adaptations and Modifications-Enhanced
HWPP: Healthy Workplace Participatory Program
HWPP-E: Healthy Workplace Participatory Program - Educators
IDEAS: Intervention Design and Analysis Scorecard
SD: Standard Deviation
TWH: *Total Worker Health®*
URP-IR: Usage Rating Profile-Intervention
